# Small bowel adenocarcinoma as a complication of celiac disease: clinical and diagnostic features

**DOI:** 10.1186/s12876-019-0964-6

**Published:** 2019-03-27

**Authors:** Giacomo Caio, Umberto Volta, Francesco Ursini, Roberto Manfredini, Roberto De Giorgio

**Affiliations:** 1 Department of Medical Sciences, University of Ferrara, St. Anna Hospital, Via Aldo Moro, 8, 44124 Ferrara, Cona Italy; 2 Mucosal Immunology and Biology Research Center and Celiac Center, Massachusetts General Hospital- Harvard Medical School, Boston, MA USA; 3 Department of Medical and Surgical Sciences, University of Bologna, Bologna, Italy

**Keywords:** Celiac disease, Diagnostic work-up, Histopathology, Overall survival, Small bowel adenocarcinoma, Treatment options

## Abstract

**Background:**

Small bowel adenocarcinoma (SBA) is a rare neoplasm, which can occur in a sporadic form or can be associated with a number of predisposing conditions such as hereditary syndromes and immune-mediated intestinal disorders, e.g. celiac disease (CD). However, the features of SBA in the context of CD remain only partly understood. This study was aimed to show the main clinical features, diagnostic procedures and management options of SBA cases detected in a large cohort of celiac patients diagnosed in a single tertiary care center.

**Methods:**

We retrospectively reviewed all the SBA cases detected in a cohort of 770 CD patients (599 females; F / M ratio: 3.5:1; median age at diagnosis 36 years, range 18–80 years), diagnosed at the Celiac Disease Referral Center of our University Hospital (Bologna, Italy) from January 1995 to December 2014.

**Results:**

Five (0.65%) out of our 770 CD patients developed SBA. All of them were female with a mean age of 53 years (range 38–72 years). SBA, diagnosed at the same time of the CD diagnosis in three cases, was localized in the jejunum in four cases and in the duodenum in one case. The clinical presentation of SBA was characterized by intestinal sub-occlusion in two cases, while the predominant manifestation of the remaining three cases was iron deficiency anaemia, abdominal pain and acute intestinal obstruction, respectively. All the patients were referred to surgery, and three cases with advanced stage neoplasia were also treated with chemotherapy. The overall survival rate at 5 years was 80%.

**Conclusions:**

Although in a limited series, herein presented CD-related SBA cases were characterized by a younger age of onset, a higher prevalence in female gender and a better overall survival compared to sporadic, Crohn- and hereditary syndrome-related SBA.

## Background

The small bowel accounts for more than 75% of the whole gastrointestinal tract and 90% of its mucosal surface. Nonetheless, the small bowel adenocarcinoma (SBA) is an extremely rare neoplasia, with an incidence of 5.7 cases per million people [1]. In Europe the estimated rate of new cases per year is 3600, with a median age in the seventh decade of life [2]. Similar findings were described in the US population, with 5300 new cases and 1100 deaths per year [3]. The duodenum is the most common localization for this neoplasia accounting for 55–82% of cases, followed by jejunum (11–25%) and ileum (7–17%) [2, 4, 5]. SBA can be detected in patients without any associated intestinal disorder (i.e., sporadic SBA) or it can occur in association with predisposing conditions, including hereditary conditions, e.g. familial adenomatous polyposis, Lynch syndrome Peutz-Jeghers syndrome and others, and immune-related intestinal disorders (i.e., Crohn’s disease and celiac disease, CD) [2, 6–8].

The association between CD and SBA was first described more than 40 years ago [9] and, in contrast to the general population, CD patients have a markedly increased risk of developing SBA. Nowadays it is largely agreed that a delay in CD diagnosis and a low compliance with gluten free diet (GFD) are two major factors leading to a 60–80 fold relative risk of developing SBA [10–12]. Another study involving a metanalysis of 79,000 CD patients estimated an odd ratio of 14.41 for developing SBA [13]. In the last years the incidence of SBA is increasing according to the data from the surveillance epidemiology and end results (SEER) showing an overall incidence of 22.7 cases per million in the US population in 2004 [14]. Similarly, the prevalence of CD is spreading worldwide, accounting for more than 1% of the general population [15].

The link between SBA and CD has been explored in a few case reports implying that a variety of putative mechanisms, e.g., chronic inflammation, increased permeability to cancerogenic factors, malabsorption of anti-cancerogenic substances (e.g., vitamins) as well as impaired immune surveillance can contribute to the pathogenesis of SBA arising from CD [2, 16]. As most intestinal tumors, also SBA may recognize the classic ‘adenoma-to-carcinoma’ sequence [17], although this hypothesis is still debated. In the present study, we aimed to provide physicians with the main clinical features, diagnostic procedures and management options of our cases of SBA detected in a large cohort of CD patients observed in a single tertiary care center in a time-frame of 20 years. Also, the information derived from this study should aid an early recognition of a very aggressive tumor usually associated with a poor quality of life and a significantly reduced life expectancy for CD patients.

## Methods

From January 1995 to December 2014, 770 CD patients (599 females; F / M ratio 3.5:1, median age at diagnosis 36 years, range 18–80 years) were diagnosed at the Celiac Disease Referral Center of the St. Orsola-Malpighi University Hospital (Bologna, Italy). The CD diagnosis was made according to duodenal histopathology, serology and Human Leukocyte Antigen (HLA) genotyping (HLA-DQ2 -DQA1*05 and DQB1*02 with detection of homozygosity and HLA-DQ8 - DQB1*0302). Serological testing was based on the detection of tissue transglutaminase (tTGA) and anti-endomysial (EmA) antibodies of IgA class. In cases with selective IgA deficiency, tTGA or deamidated gliadin peptides (DGP) of IgG class were assayed (Eurospital, Trieste, Italy). A least *n* = 4 biopsies were taken both from duodenal bulb (*n* = 2) and second duodenal portion (*n* = 2). All cases of SBA occurring in this cohort of 770 CD patients were retrospectively investigated. Once a case of CD-related SBA was identified, all available data, regarding CD diagnosis, diagnostic work-up, histopathology, treatment and survival rate were obtained from the hospital digital database.

A simplified International Review Board approval by the Ethics Committee of the St. Orsola-Malpighi Hospital was obtained and each patient (or a family member in case of exitus) signed the consent form for publication.

## Results

We identified 5 cases of SBA out of 770 CD patients (0.65%). All SBA were diagnosed in female patients with a mean age of 53 years (range 38–72 years). The SBA diagnosis was established at the same time of the CD diagnosis in three cases. The CD clinical picture at diagnosis was characterized by classical symptoms (i.e., chronic diarrhea and malabsorption) in two cases, while the other three cases showed a non-classical CD phenotype characterized by iron deficiency anaemia, constipation and abdominal pain. In four out of five cases the CD serology (tTGA and EmA) was positive at high titer (mean tTGA titer: 91 AU, range 86–114 AU; mean EmA titer 1:160, range 1:80–1:320). The HLA genotyping showed DQ2 positivity in all cases, of whom only one with DQ2 homozigosity. The duodenal histopathology at CD diagnosis was consistent with a subtotal villous atrophy (grade 3C according to Marsh-Oberhüber classification) in four cases and a mild villous atrophy (grade 3A) in one. The most frequent localization of the SBA was the jejunum in four cases, whereas only one case was localized to the duodenum. The clinical presentation of SBA was characterized by intestinal sub-occlusion in two cases, while the predominant manifestation of the remaining three cases was iron deficiency anaemia, abdominal pain and acute intestinal obstruction, respectively (Table 1).

**Table 1:** Clinical features of patients with small bowel adenocarcinoma in the celiac cohort

Case #	Sex	HLA	Age at CD diagnosis (yrs)	Age at SBA diagnosis (yrs)	SBA localization	TNM at diagnosis	Presentation	Key diagnostic exam(s) for SBA	Chemotherapy	Survival at 6 years
1	F	DQ2	68	72	Jejunum	T3N0M0	Acute intestinal obstruction	Abdominal X-ray; double contrast upper GI radiography; Exploratory laparotomy	None	Alive
2	F	DQ2 homozygosis	34	69	Jejunum	T2N0M0	Iron deficiency anaemia	VCE; DBE	None	Alive
3	F	DQ2	46	46	Jejunum	T4N2M2	Intestinal sub-occlusion	Small bowel loop ultrasound; CT enteroclysis	FOLFOX + Bevacizumab	Dead after 13 months
4	F	DQ2	38	38	Jejunum	T3N2M0	Intestinal sub-occlusion	CT scan; PET; DBE	FOLFOX	Alive
5	F	DQ2	40	40	Duodenum	T3N2M0	Abdominal pain	OGD	FOLFOX + FOLFIRI	Dead after 63 months

Abbreviations: *HLA* human leukocyte antigens, *CD* celiac disease, *SBA* small bowel adenocarcinoma, *F* female, *TNM* tumor, nodes, metastasis classification, *OGD* oesophago-gastro-duodenal endoscopy, *GI* gastrointestinal, *CT* computed tomography, *PET* positron emission tomography, *DBE* double balloon enteroscopy, *FOLFOX* folinic acid (leukovorin), 5-fluorouracil, oxaliplatin, *FOLFIRI* folinic acid (leukovorin), 5-fluorouracil, irinotecan, *VCE* video capsule endoscopy

Notably, the SBA diagnosis was made in four out of five cases before surgery via a thorough diagnostic work-up including small bowel loop ultrasound (SBLUS), videocapsule (VCE), double-balloon enteroscopy (DBE), computed tomography enteroclysis (CTE), magnetic resonance enteroclysis (MRE), ^18^fluorodeoxyglucose positron emission tomography (^18^FDG-PET). The only case in which the SBA diagnosis was reached at laparotomy was before 2004 when high sensitive imaging techniques were not available in our hospital.

The histological evaluation showed the presence of a poor differentiated, high-grade carcinoma (G3-G4) in three cases, associated to lymph nodes and metastasis to liver, peritoneum and pancreas in two of them (Table 2). The other two cases showed low-grade (G1-G2) intestinal type adenocarcinoma with tubular and cribriforming glands, penicillate nuclei with pseudo-stratification and brush border, without lymph node involvement or distant metastases (Table 2).

**Table 2:** Histopathological features of patients with small bowel adenocarcinoma in the celiac cohort

Case #	Depth of infiltration	Lymph nodes	Metastasis	SBA histopathological grading	CD histopathology
1	Muscularis propria	Negative	None	Well differentiated (G2)	Subtotal villous atrophy (3c)
2	Submucosa	Negative	None	Well differentiated (G2)	Subtotal villous atrophy (3c)
3	Serosa	Positive	Liver, peritoneum	Poorly differentiated (G4)	Mild villous atrophy (3a)
4	Perivisceral fat	Positive	none	Poorly differentiated (G4)	Subtotal villous atrophy (3c)
5	Muscularis propria	Positive	Pancreas, peritoneum	Poorly differentiated (G4)	Subtotal villous atrophy (3c)

All the patients were referred to surgery, and three cases with advanced stage neoplasia were also treated with chemotherapy, using 5-fluorouracil / leukovorin / oxaliplatin (FOLFOX); this treatment was associated with the humanized anti-vascular endothelial growth factor (VEGF) monoclonal antibody, bevacizumab, in one case. The overall survival rate at 5 years was 80%, since only one patient died after 13 months due to the progression of SBA. Another patient died, but after 63 months from the diagnosis with a 6-year overall survival of 60%. Both these two latter cases were high-stage (IV and IIIB, respectively), high-grade, poorly differentiated SBA.

### Case series

#### Case no. 1

The patient, a 72-year old white female, was diagnosed CD at the age of 68. The clinical presentation of CD was non-classical, characterized by an iron deficiency anaemia and constipation from her youth. The upper endoscopy at the time of CD diagnosis showed a subtotal villous atrophy (3C) and a *Helicobacter pylori* gastritis, which was eventually eradicated. CD serology including tTGA and EmA was positive at high titer. Despite more than 2 years of a strict gluten free diet (GFD) with negativization of CD antibodies, the patient continued to experience severe iron deficiency anaemia (Hb 8.0 g/dl). In order to investigate the severe anaemia, a fecal occult blood (FOB) test was performed, with a positive result. An oesophago-gastro-duodenal endoscopy (OGD) was repeated with no remarkable findings and the histological examination of duodenal biopsies showed an increased number of intraepithelial lymphocytes (IEL) (grade 1) consistent with a CD in GFD. Moreover, the patient underwent colonoscopy, small bowel follow through, abdominal ultrasonography and computed tomography (CT) scan with no major findings. Meanwhile the patient was treated with iron infusions with success, and a new determination of FOB test turned to be negative. All blood tests were normal including white blood count (WBC), platelets, erythrocyte sedimentation rate (ESR), total serum protein, liver, kidney, thyroid function tests and carcinoembryonic antigen (CEA). After few months, the patient was admitted at the emergency department for the onset of an acute intestinal obstruction with vomiting. An abdominal X-ray showed a gastric air-fluid level and the routine blood tests indicated a mild iron deficiency anaemia (Hb 11.8 g/dl, mean cellular volume 78 fl, platelets 528.000/mm3, ferritin 10 ng/ml). A nasogastric tube was placed and drained about 4 l of bilious fluids. A double contrast upper gastrointestinal (GI) radiography showed a stenosis localized at a distance of 10–15 cm from Treitz. The patient underwent an exploratory laparotomy with a jejunal resection and local lymphadenectomy. The histological characterization of the stenotic mass showed a low-grade (G2) intestinal type adenocarcinoma with tubular and cribriforming glands, penicillate nuclei with pseudo-stratification and infiltration of the *muscularis propria*, without lymph node invasion (pT3N0). Metastases were excluded by a total body contrast enhanced CT scan, thus the patient was not treated with an adjuvant chemotherapy. After 12 years of follow-up, the patient is doing well and is free from tumor recurrences.

#### Case no. 2

The patient, a 69-year old white female, was diagnosed CD at the age of 34. She had a typical presentation of CD characterized by chronic diarrhea, weight loss, malabsorption, and recurrent miscarriages. The diagnosis was made during the 70s of the last century using a Crosby-Watson capsule, but despite this early diagnosis, the patient did not adhere to a strict GFD and follow-up program. Thirty-five years after the CD diagnosis, the patient was referred to our attention due to the exacerbation of abdominal pain, vomiting and weight loss. The CD diagnosis was confirmed by the presence of tTGA and EmA at high titer along with a subtotal villous atrophy (grade 3C) at the duodenal biopsy. GFD yielded only a partial clinical response. In the following months, the onset of a severe anaemia (Hb 6.0 g/dl) led us to suspect an occult gastrointestinal bleeding, confirmed by a FOB test. Both colonoscopy and OGD were completely normal. The patient underwent VCE examination to exclude possible bleeding sources in the small bowel, which identified a focal ulceration narrowing the jejunum at approximately 50 cm after Treitz. A DBE reached the lesion and the biopsy revealed a low-grade (G2) intestinal type adenocarcinoma. A surgical resection with local lymphadenectomy was performed and the histopathological analysis on the removed material confirmed the presence of an infiltrating SBA invading the submucosa, although sparing the *muscularis propria*. No neoplastic lymph nodes were found (0/20), thus the lesion was classified as pT2N0. After 10 years of follow-up the patient is in good health without tumor recurrences.

#### Case no. 3

The patient, a 46-year old female, was diagnosed to have SBA after only 4 months from the CD diagnosis. The clinical picture was characterized by a non-classical phenotype with a long history of constipation, menstrual irregularities with a miscarriage in the first trimester, enamel defects and asthenia. The duodenal biopsy was consistent with mild villous atrophy (grade 3A) and the serology showed a positivity for tTGA and EmA at medium titer. Other routine blood tests were normal (i.e., Hb 14.8 g/dl, ferritin 108 ng/dl, albumin 3.9 g/dl). GFD was started right after diagnosis, but after 2 months the patient experienced a significant weight loss, vomiting episodes and severe asthenia. The lack of clinical response, the late CD diagnosis and worsening of symptoms prompted us to investigate the patient for possible complications. CD serology resulted negative indicating a good compliance to GFD and blood tests showed WBC 10.800/mm^3^, (hemoglobin) Hb 13.1 g/dl, platelets 640.000/mm^3^, aspartate aminotransferase (AST) 81 U/L (n.v. < 32 U/L), alanine aminotransferase (ALT) 45 U/L (n.v. < 31 U/L), gamma glutamyl transferase (GGT) 22 U/L (n.v. < 36 U/L), alkaline phosphatase (ALP) 371 U/L (n.v. < 280 U/L), lactate dehydrogenase (LDH) 580 U/L (< 480 U/L), ESR 20 mm (n.v. < 17), albumin 3,2 g/dl, CEA 17.1 ng/ml (< 5 ng/ml) and Ca19.9792 (n.v. < 37). A contrast (Sonovue) enhanced SBLUS revealed small bowel loop dilatation and the presence of a dyshomogeneous mass (20 × 24 mm) in the left side of the abdomen characterized by a contrast enhancement in the arterial phase and a quick wash-out in the portal venous phase, a pattern suggestive of a neoplastic lesion. Also, this exam revealed the existence of multiple liver metastases. A CTE with oral polyethylene glycol and a MRE confirmed a pathologic thickness of the small bowel mucosa causing a partial obstruction of the lumen, localized at about 20 cm from Treitz. The patient underwent a laparotomy revealing a massive peritoneal carcinosis (T4N2M2). The histological evaluation revealed a high-grade (G4) poorly differentiated adenocarcinoma with serosa infiltration. A FOLFOX palliative chemotherapy was prescribed, along with bevacizumab; nonetheless, the patient died 13 months after SBA diagnosis because of the advanced neoplastic disease.

#### Case no. 4

This patient was a 36-year old woman, the youngest patient in our series. The patient complained of new onset abdominal pain and constipation. Her family doctor recommended a colonoscopy, which resulted completely normal, and an OGD revealing *Helicobacter pylori* negative chronic gastritis. Notably, no duodenal biopsies were taken during this exam. All the blood tests were normal including Hb, WBC, platelets, ESR, total serum protein, liver, kidney, thyroid function tests and CEA. Thus, a diagnosis of constipation predominant irritable bowel syndrome (IBS-C) was established. Twenty months after the IBS-C diagnosis, the patient (turning 38 years) was admitted to the emergency department of our hospital for the onset of vomiting, weight loss (5 Kg in 1 month) and worsening of chronic abdominal pain. An abdominal X-ray showed only coprostasis with absence of air-fluid levels. WBC count was 10.301/mm^3^ (78% neutrophils), Hb 12.6 g/dl, mean cellular volume 71.9 fl, platelets 413.000/mm^3^, ferritin 10 ng/ml (n.v. 15–155 ng/ml), ESR 75 mm (< 17), C-reactive protein 1.30 mg/dl (< 0.80) and hyperuricemia (8.0 mg/dl; n.v. < 5.7 mg/dl), total serum protein, liver, kidney, thyroid function tests were normal. During the hospitalization a CD serological screening demonstrated high titer tTGA and EmA, whereas a duodenal biopsy showed the presence of subtotal villous atrophy (grade 3C). Despite GFD, the persistence of vomiting episodes led us to investigate the patient with a contrast enhanced abdominal CT scan detailing a 5-cm long thickening (up to 7 mm in depth) of a proximal jejunal loop and the presence of multiple enlarged mesenterial lymph nodes (up to 3 × 2.2 cm). Because of the unclear definition of the identified lesion, a MRE and a ^18^FDG-PET were performed. The imaging techniques showed the presence of pathological contrast enhancement of the jejunal thickening (Standardized Uptake Value, SUV max 17) and mesenterial lymph nodes (SUV max 21) suggestive of intestinal lymphoma. However, CEA and Ca19.9 positivity (17 ng/dl, n.v. < 5; and 792 ng/dl, n.v. < 37, respectively), along with normal LDH and beta2 microglobulin, argued against the diagnosis of lymphoma. Thus, the patient underwent a DBE with biopsies, which disclosed a poorly differentiated, high-grade (G4), intestinal type adenocarcinoma. The patient was operated on with jejunal resection, omentectomy and local lymphadenectomy. The analysis of the resected intestinal tract confirmed the diagnosis of infiltrating SBA with perivisceral adipose tissue involvement and 16/20 lymph nodes involved by tumor (pT3N2). Notably, the histological post-surgical evaluation revealed a high-grade intraepithelial neoplastic focus adjacent to the primary lesion. Due to the presence of a stage IIIB neoplasia, an adjuvant chemotherapy with FOLFOX was prescribed. After 5 years of follow-up, the patient is still alive with an active neoplastic disease.

#### Case no. 5

The patient, a 40-year old white female was referred to OGD (performed in other center) for an epigastric pain. The upper endoscopy showed a neoplastic lesion of the second duodenal portion defined as a high-grade, poorly differentiated adenocarcinoma at histology. Thus, the patient was surgically treated with a duodeno-cephalo-pancreasectomy. Eighteen days post-surgery, the patient had a complication characterized by an intestinal invagination, which required a re-intervention. The histological evaluation of the resected mass confirmed the presence of a poorly differentiated, high-grade (G4) adenocarcinoma infiltrating the *muscularis propria* along with the positivity of 10/20 lymph nodes (pT3N2). After the second intervention, the patient developed a diarrhea resistant to classic anti-diarrheal drugs. The patient started an adjuvant chemotherapy with FOLFOX, which was associated with worsening of diarrhea (up to 20 bowel movements / day). The patient was referred to our Center and started a thorough diagnostic work-up aimed to define the origin of her neoplastic condition. We decided to perform a serological screening for CD, which indicated high titer positivity of tTGA and EmA and the jejunal biopsy revealed a subtotal villous atrophy (3C). GFD promptly started by the patient led to a marked improvement of the diarrhea. The first and second year of follow-up were regular, with antibody negativization and mucosal regrowth (Marsh 1). At the third year, a low-titer positivity for tTGA was detected and a control biopsy revealed a Marsh 3A lesion. This phenomenon was explained by the voluntary introduction of gluten. In the same year, a follow-up CT scan revealed a reoccurrence of neoplastic disease involving the pancreas and peritoneum. A second line chemotherapy with 5-fluorouracil / leukovorin / irinotecan (FOLFIRI) was attempted, but the patient died 5 years and 3 months after the SBA diagnosis.

## Discussion

The results of the present study expand the limited information available on the association between CD and SBA, a complication which occurs in misdiagnosed celiacs or in patients not fully compliant to GFD [17–20]. Because of the limited number of studies, it is unknown whether CD-related SBA differs from sporadic SBA and SBA cases associated to intestinal disorders other than CD, including Crohn’s, Peutz-Jeghers, Lynch syndrome and familial adenomatous polyposis [2, 6–8]. Our analysis, including one of the largest series of CD-related SBA, clearly demonstrated that the SBA onset in CD patients exhibits peculiar clinical and histological features. In order to better highlight these peculiarities, the findings emerged from our CD-related SBA cases have been compared to those with sporadic SBA and SBA associated to the other (non CD-related) intestinal disorders so far reported in the literature [2, 6–8].

In our cohort of 770 CD patients, SBA in CD showed a prevalence of 0.65%, which indicates a much higher frequency of SBA compared to that estimated in the general population (5.7 cases / 1.000.000 people) [1]. The higher prevalence observed in this study likely reflects a possible selection bias resulting from patients investigated in a tertiary referral center. In this line, another remarkable information emerging from our study is that SBA was exclusively detected in female CD patients, a finding in contrast with data reporting a slightly higher prevalence of SBA and other small bowel neoplasms in male vs. female gender [3, 21]. Also, the age of SBA onset in CD patients was lower than that reported in the literature for SBA occurring in non-CD patients (53 vs. 63 years old) [3, 21, 22]**.** In three out of five cases of our series the diagnosis of CD was coincident to that of SBA in a relatively young age (mean age: 41.3 years), a finding suggesting that a thorough diagnostic work-up should be advised in CD patients presenting with alarm symptoms / signs including abdominal pain, nausea, vomiting, weight loss and detection of FOB as emerged in case n. 3 and n. 4.

Previous studies indicate that almost all CD-related SBA cases showed a long history of CD characterized by classical symptoms, i.e. diarrhea and malabsorption [9, 17, 18]. Conversely, our study showed that SBA can occur in CD patients with both classical and non-classical symptom profile. SBA was slightly more frequent in CD patients with non-classical symptom profile (characterized by iron-deficiency anaemia, constipation, recurrent miscarriages, menstrual irregularity and enamel defects) than those with a classical malabsorption syndrome with diarrhea and weight loss (3 vs. 2 cases, respectively). The risk of developing SBA and other CD associated diseases appears to be higher in patients with a long history of misdiagnosed / untreated CD [17–20, 23]. A paradigmatic example of such concept is given by patient n. 2 who did not adhere to GFD until SBA occurred 35 years later from the initial diagnosis of CD. The possible onset of SBA in CD patients with non-classical and less severe extraintestinal manifestations should alert physicians to investigate any patients with CD even those without intestinal alarm symptoms. Such strategy is mandatory in those cases with a late CD diagnosis (e.g. after 45 year-old) or with a poor compliance to GFD. Unfortunately, no international guidelines are so far available regarding management of special subsets of CD patients, such as those diagnosed after the fifth decade of life, with a long history of undiagnosed CD and continuous gluten ingestion [24, 25]. Moreover, it is mandatory that physicians, endoscopists and pathologists rule out CD when detecting a patient with SBA or small intestine adenomas, avoiding a significant delay in CD diagnosis as described in case n. 5.

Our study provided further insight for the genetic pattern of CD-related SBA. Positivity for HLA-DQ2 and/or DQ8 is crucial for the development of CD and several studies demonstrated that the positivity for HLA-DQ2 homozigosity is closely related to CD complications, such as refractory CD and small intestinal lymphoma [24–26]. Notably, all CD-related SBA cases investigated in the present study were HLA-DQ2 positive, but only one case displayed homozygosis. Moreover, in our study none of the CD-related SBA patients had a previous diagnosis of refractory CD. Therefore, SBA and intestinal lymphoma occurring in CD patients differ from each other, since the former does not show any correlation with HLA-DQ2 homozigosity and it is not preceded by refractory CD, which, conversely, is typical of intestinal lymphoma [26].

Another new finding emerging from the present study is about the SBA localization in the intestinal tract of CD patients. In contrast with current literature, reporting a predominant localization of all the SBA subtypes (sporadic, associated with hereditary syndromes and immune mediated disorders) in the duodenum in up to 60% of cases, the CD-related SBA is localized in the vast majority of cases in the jejunum [2, 6–8, 16]. In contrast with Crohn-related SBA where an adenoma-carcinoma sequence has been established [27], this pathogenetic progression remains doubtful in CD-related SBA. A case series from Rampertrab et al. proposed the adenoma-to-carcinoma evolution by describing three cases of CD with duodenal adenoma, although no other studies could eventually confirm this hypothesis [17]. Notably, in the present paper, we did find in case n. 4 a flat, high-grade intraepithelial neoplasia, adjacent to the primary invasive carcinoma. Although marginal, this evidence suggests the possibility that a flat dysplasia of the mucosa extending from the tumor margin or distant from the primary carcinoma may be the primary lesion initiating the sequence leading to an established carcinoma.

Based on the results of our study, the 5-year survival of CD-related SBA appears to be 80% of cases, a figure which is significantly better than that of sporadic SBA (14–33%), Crohn-related SBA (38%) and small intestinal lymphoma (8–20%) [2, 16, 28, 29]. Notably, it is worth pointing out that cases with advanced CD-related SBA (including the three cases with N2) showed an overall long survival. In this respect, compared to the sporadic one, the CD-related SBA might be considered a less aggressive adenocarcinoma [2, 16]. An early diagnosis of CD-related SBA is therefore mandatory for an optimal management of affected patients. Best therapeutic options include radical surgery (segmental enterectomy) and chemotherapy [30]. No benefit has been demonstrated for post-surgery adjuvant chemotherapy in patients with any form of SBA, whereas retrospective studies showed that chemotherapy prolonged overall survival in patients with advanced SBA [31, 32]. Therapeutic regimens for metastatic SBA include FOLFOX (a combination of oxaliplatin, 5-fluorouracil and leukovorin), XELOX (capecitabine and oxaliplatin) and FOLFIRI (irinotecan, 5-fluorouracil and leukovorin) with / without bevacizumab, a humanized anti-vascular endothelial growth factor (VEGF) monoclonal antibody [33].

In recent years the availability of improved diagnostic techniques, such as SBLUS, VCE, DBE, MRE, CTE and ^18^FDG-PET, has allowed an early diagnosis, i.e. when the neoplasm is still localized without metastases, in the majority of cases. However, a recent population-based study showed that the diagnostic implementation of new techniques did not yield significant advantage in terms of early diagnosis and better outcome [34]. In patients with CD-related SBA diagnosis, an aggressive approach is justified because of the good 5-year survival. On the other hand, every patient undergoing surgery for SBA should be tested to rule out CD through an accurate histopathological evaluation of the resected material (particularly looking for mucosal atrophy in tumor-free margins), possibly preceded by intraoperative duodenal biopsies, and serological tests [24, 25, 35]. This approach is mandatory as in most cases CD diagnosis is established at the same time or, more commonly, after SBA detection. In this respect, the timely institution of GFD might reduce the post-surgical intestinal morbidity. In our series, the only patient experiencing post-surgical complications was case n. 5, who did not start GFD due to a misdiagnosis of CD. Whether GFD plays a role in reducing CD-related risk of SBA remains an open issue eagerly awaiting further investigation.

## Conclusions

We do recognize that this study has some limits, such as the retrospective design, which may hamper the interpretation of our results, in particular those regarding the outcome, because of the small number of patients. The relatively favorable prognosis of CD-related SBA in our series may reflect the cautious follow-up of CD patients in the healthcare system. However, given the few publications on this topic, five cases of SBA in a cohort of 770 CD patients represent an initial basis to better understand CD-related SBA and its many clinical aspects. So far, available studies pooled together various cases, i.e. sporadic SBA along with SBA related to a number of conditions, including CD. In this paper, we sought to dissect out the clinical, diagnostic and outcome features of CD-related SBA from all other cases. Taken together our data contributed to define some findings of SBA in CD and therefore to expand current knowledge on these patients. Further international multicenter studies are awaited to understand why SBA shows different clinical features and prognosis in different patient subsets.

## Abbreviations

^18^FDG-PET: ^18^fluorodeoxyglucose positron emission tomography
ALP: Alkaline phosphatase
ALT: Alanine aminotransferase
AST: Aspartate aminotransferase
Ca19.9: Carbohydrate antigen 19.9
CD: Celiac disease
CEA: Carcinoembryonic antigen
CT: Computed tomography
CTE: Computed tomography enteroclysis
DBE: Couble-balloon enteroscopy
DGP: Deamidated gliadin peptides
EmA: Anti-endomysial antibodies
ESR: Erythrocyte sedimentation rate
FOB: Fecal occult blood
FOLFIRI: 5-fluorouracil / leukovorin / irinotecan
FOLFOX: 5-fluorouracil / leukovorin / oxaliplatin
GFD: Gluten free diet
GGT: Gamma glutamyl transferase
GI: Gastrointestinal
Hb: Hemoglobin
HLA: Human leukocyte antigen
IEL: Intraepithelial lymphocytes
LDH: Lactate dehydrogenase
MRE: Magnetic resonance enteroclysis
OGD: Oesophago-gastro-duodenal endoscopy
SBA: Small bowel adenocarcinoma
SBLUS: Small bowel loop ultrasound
SEER: Surveillance epidemiology and end results
SUV: Standardized Uptake Value
tTGA: tissue transglutaminase antibodies
VCE: Videocapsule endoscopy
VEGF: Vascular endothelial growth factor
WBC: White blood count
XELOX: Capecitabine and oxaliplatin

## References

[1] Neugut AI, Jacobson JS, Suh S, Mukherjee R (1998). Arber. The epidemiology of cancer of the small bowel. Cancer Epidemiol Biomark Prev.

[2] Aparicio T, Zaanan A, Svrcek M, Laurent-Puig P, Carrere N, Manfredi S (2014). Small bowel adenocarcinoma: epidemiology, risk factors, diagnosis and treatment. Dig Liver Dis.

[3] Haselkorn T, Whittemore AS, Lilienfeld DE (2005). Incidence of small bowel cancer in the United States and worldwide: geographic, temporal, and racial differences. Cancer Causes Control.

[4] Neugut AI, Marvin MR, Rella VA, Chabot JA (1997). An overview of adenocarcinoma of the small intestine. Oncology..

[5] Abraham J, Gulley JL, Allegra CJ (2010). Handbook of clinical oncology.

[6] Palascak-Juif V, Bouvier AM, Cosnes J, Flourié B, Bouché O, Cadiot G (2005). Small bowel adenocarcinoma in patients with Crohn’s disease compared with small bowel adenocarcinoma de novo. Inflamm Bowel Dis.

[7] Shaukat A, Virnig DJ, Howard D, Sitaraman SV, Liff JM, Lederle FA (2011). Crohn’s disease and small bowel adenocarcinoma: a population-based case–control study. Cancer Epidemiol Biomark Prev.

[8] Piton G, Cosnes J, Monnet E, Beaugerie L, Seksik P, Savoye G (2008). Risk factors associated with small bowel adenocarcinoma in Crohn’s disease: a case-control study. Am J Gastroenterol.

[9] Kenwright S (1972). Coeliac disease and small bowel carcinoma. Postgrad Med J.

[10] Silano M, Volta U, Mecchia AM, Dessì M, Di Benedetto R, De Vincenzi M (2007). Delayed diagnosis of coeliac disease increases cancer risk. BMC Gastroenterol.

[11] Green PHR, Stavropoulos SN, Panagi SG, Goldstein SL, Mcmahon DJ, Absan H, Neugut AI (2001). Characteristics of adult celiac disease in the USA: results of a national survey. Am J Gastroenterol.

[12] Swinson CM, Slavin G, Coles EC, Booth CC (1983). Coeliac disease and malignancy. Lancet..

[13] Han Y, Chen W, Li P, Ye J (2015). Association between coeliac disease and risk of any malignancy and gastrointestinal malignancy: a meta-analysis. Medicine (Baltimore).

[14] Surveillance, Epidemiology, and End Results Program, 1975-2005, Division of Cancer control and population sciences, National Cancer Institute, 2008.

[15] Volta U, Caio G, Stanghellini V, De Giorgio R (2014). The changing clinical profile of celiac disease: a 15-year experience (1998-2012) in an Italian referral center. BMC Gastroenterol.

[16] Vanoli A, Di Sabatino A, Furlan D, Klersy C, Grillo F, Fiocca R (2017). Small bowel carcinomas in coeliac or Crohn's disease: Clinico-pathological, molecular, and prognostic features. A study from the small bowel Cancer Italian consortium. J Crohns Colitis.

[17] Rampertab SD, Forde KA, Green PH (2003). Small bowel neoplasia in coeliac disease. Gut..

[18] Holmes GK, Dunn GI, Cockel R, Brookes VS (1980). Adenocarcinoma of the upper small bowel complicating coeliac disease. Gut..

[19] Javier J, Lukie B (1980). Duodenal adenocarcinoma complicating celiac sprue. Dig Dis Sci.

[20] Farrell DJ, Shrimankar J, Griffin SM (1991). Duodenal adenocarcinoma complicating coeliac disease. Histopathology..

[21] Bilimoria KY, Bentrem DJ, Wayne JD, Ko CY, Bennett CL, Talamonti MS (2009). Small bowel cancer in the United States: changes in epidemiology, treatment, and survival over the last 20 years. Ann Surg.

[22] Terada T (2012). Malignant tumors of the small intestine: a histopathologic study of 41 cases among 1,312 consecutive specimens of small intestine. Int J Clin Exp Pathol.

[23] Volta U, Caio G, Tovoli F, De Giorgio R (2013). Gut-liver axis: an immune link between celiac disease and primary biliary cirrhosis. Expert Rev Gastroenterol Hepatol.

[24] Ludvigsson JF, Bai JC, Biagi F, Card TR, Ciacci C, Ciclitira PJ (2014). Diagnosis and management of adult coeliac disease: guidelines from the British Society of Gastroenterology. Gut..

[25] Rubio-Tapia A, Hill ID, Kelly CP, Calderwood AH, Murray JA (2013). American College of Gastroenterology. ACG clinical guidelines: diagnosis and management of celiac disease. Am J Gastroenterol.

[26] Biagi F, Gobbi P, Marchese A, Borsotti E, Zingone F, Ciacci C (2014). Low incidence but poor prognosis of complicated coeliac disease: a restrospective multicentre study. Dig Liver Dis.

[27] Simpson S, Traube J, Riddell RH (1981). The histologic appearance of dysplasia (precarcinomatous change) in Crohn’s disease of the small and large intestine. Gastroenterology..

[28] Van de Water JM, Cillessen SA, Visser OJ, Verbeek WH, Meijer CJ, Mulder CJ. Enteropathy associated T-cell lymphoma and its precursor lesions. Best Pract Res Clin Gastroenterol. 2010;24:43–56. 10.1016/j.bpg.2009.11.002 20206108

[29] Nijeboer P, Malamut G, Mulder CJ, Cerf-Bensussan N, Sibon D, Bouma G (2015). Enteropathy-associated T-cell lymphoma: improving treatment strategies. Dig Dis.

[30] Howe JR, Karnell LH, Menck HR, Scott-Conner C (1999). The American College of Surgeons Commission on Cancer and the American Cancer Society. Adenocarcinoma of the small bowel: review of the National Cancer Data Base, 1985-1995. Cancer..

[31] Halfdanarson TR, McWilliams RR, Donohue JH, Quevedo JF (2010). A single-institution experience with 491 cases of small bowel adenocarcinoma. Am J Surg.

[32] Fishman PN, Pond GR, Moore MJ, Oza A, Burkes RL, Siu LL (2006). Natural history and chemotherapy effectiveness for advanced adenocarcinoma of the small bowel: a retrospective review of 113 cases. Am J Clin Oncol.

[33] Zaanan A, Gauthier M, Malka D, Locher C, Gornet JM, Thirot Bidault A (2011). Second-line chemotherapy with fluorouracil, leucovorin and irinotecan (FOLFIRI regimen) in patients with advanced small bowel adenocarcinoma after failure of first line platinum-based chemotherapy: a multicenter AGEO study. Cancer..

[34] Legué LM, Bernards N, Gerritse SL, van Oudheusden TR, de Hingh IH, Creemers GM, Ten Tije AJ, Lemmens VE (2016). Trends in incidence, treatment and survival of small bowel adenocarcinomas between 1999 and 2013: a population-based study in the Netherlands. Acta Oncol.

[35] Volta U, Caio G, Boschetti E, Giancola F, Rhoden KJ, Ruggeri E (2016). Seronegative celiac disease: shedding light on an obscure clinical entity. Dig Liver Dis.

